# Supplementary material for: Care-related quality of life of informal caregivers of stroke survivors: Cross-sectional analysis of a randomized clinical trial
Source: PLoS One. 2024 Oct 4;19(10):e0307930. doi: 10.1371/journal.pone.0307930 (PMC11452055; doi:10.1371/journal.pone.0307930)
Supplement: S1 Table — (DOCX) [file pone.0307930.s002.docx]

**S1 Table.** Bivariate analysis, CarerQol-7D/CarerQol-VAS with characteristics of caregivers and care receivers

|  | Association with CarerQol-7D | | Association with CarerQol-VAS | |  |
| --- | --- | --- | --- | --- | --- |
| **Characteristics of informal caregivers** | **Estimate (95% CI)** | **p-value** | **Estimate (95% CI)** | **p-value** | |
| Age, years (for each year increase) | 0.26 (-0.05, 0.56) | 0.101 | -0.01 (-0.05, 0.03) | 0.533 | |
| Sex, female | **-11.32 (-19.76, -2.88)** | **0.009** | -0.90 (-1.93, 0.14) | 0.088 | |
| Highest educational level (ref. primary studies incomplete) |  |  |  |  | |
| Primary studies | -6.83 (-20.85, 7.20) | 0.337 | -0.11 (-1.83, 1.60) | 0.897 | |
| Secondary | -12.96 (-26.87, 0.95) | 0.067 | -0.07 (-1.77, 1.64) | 0.94 | |
| Higher education | -11.46 (-27.08, 4.17) | 0.149 | 0.56 (-1.35, 2.47) | 0.564 | |
| Relationship with the care receiver (ref. Partner) |  |  |  |  | |
| Father or mother of the caregiver | -4.30 (-12.45, 3.85) | 0.299 | 0.27 (-0.74, 1.28) | 0.599 | |
| Son or daughter | 13.31 (-3.35,29.97) | 0.116 | -0.35 ( -2.42, 1.72) | 0.739 | |
| Father or mother-in-law | 4.07 (-26.10, 34.24) | 0.79 | 0.22 (-3.52, 3.97) | 0.907 | |
| Brother or sister | **24.21 (4.75, 43.67)** | **0.015** | 2.02 (-0.39, 4.44) | 0.1 | |
| No Lives with care receiver (ref. yes) | -4.31 (-14.84, 6.22 | 0.419 | 0.37 (-0.88, 1.61) | 0.559 | |
| Living together with children or grandchildren at home (ref. No) | -8,26 (-17.09, 0.56) | 0.066 | 0.47 (- 0.59, 1.53) | 0.385 | |
| **Characteristics of care receivers** | **Estimate 95% CI** | **p-value** | **Estimate 95% CI** | **p-value** | |
| Age, years (for each year increase) | 0.07 (-0.18, 0.32) | 0.586 | 0.01 (-0.02 , 0.04) | 0.703 | |
| Sex, female | 0,36 (-7.37, 8.08) | 0.927 | 0.57 (-0.36, 1.50) | 0.227 | |
| Pathological history and risk factors |  |  |  |  | |
| Hypertension | -3.31 (-11.49, 4.88) | 0.425 | -0.21 (-1.22, 0.80) | 0.678 | |
| Dyslipemia | 2.66 (-4.97, 10.29) | 0.491 | 0.52 (-0.40, 1.43) | 0.269 | |
| Current smoking | -2.92 (-12.89, 7.05) | 0.563 | 0.14 (-1.06, 1.34) | 0.82 | |
| Enolism | -10.53 (-22.68, 1.61) | 0.088 | -0.16 (-1.64, 1.32) | 0.829 | |
| Diabetes | 2.89 (-5.69, 11.46) | 0.506 | -0.37 (-1.39, 0.66) | 0.481 | |
| Ischemic heart disease | -4.76 (-16.64, 7.13) | 0.43 | 0.29 (-1.10, 1.69) | 0.677 | |
| Heart failure | 6.22 (-9.58, 22.02) | 0.438 | 1.28 (-0.62, 3.17) | 0.185 | |
| Peripheral arterial disease | 3.88 (-12.97, 20.73) | 0.649 | 0.23 (-1.81, 2.26) | 0.826 | |
| Previous TIA stroke | 0.81 (-9.54, 11.16) | 0.877 | 0.31 (-0.93 , 1.56) | 0.621 | |
| Atrial fibrillation | -5.96 (-15.05, 3.13) | 0.197 | -0.44 (-1.54, 0.66) | 0.426 | |
| Previous anticoagulation | -7.60 (-17.49, 2.29) | 0.131 | -0.39 (-1.59, 0.81) | 0.52 | |
| Baseline NIHSS | -**0.84 (-1.51, -0.17)** | **0.015** | **-0.10 (-0.18, -0.02)** | **0.013** | |
| Stroke etiology (ref. ischemic) |  |  |  |  | |
| Hemorrhagic | 4.08 (-4.80, 12.97) | 0.365 | 0.28 (-0.79, 1.35) | 0.606 | |
| Mimic | -7.58 (-27.46, 12.30) | 0.452 | -2.15 (-4.52, 0.23) | 0.076 | |
| Modified Rankin scale (ref. 1-2) |  |  |  |  | |
| 3 | -14.14 (-35.30, 7.02) | 0.189 | -1.85 (-4.17, 0.48) | 0.118 | |
| 4 | **-23.53 (-45.24, -1.82)** | **0.034** | **-2.80 (-5.20, -0.40)** | **0.023** | |
| 5 | **-32.35 (-55.24, -9.45)** | **0.006** | **-3.61 (-6.17, -1.05)** | **0.006** | |
| Barthel Index (ref. 60-100) |  |  |  |  | |
| 35 - 55 | -5.16 (-14.31, 3.99) | 0.266 | -1.04 (-2.14, 0.05) | 0.062 | |
| 0 - 35 | **-16.31 (-25.57, -7.04)** | **0.001** | **-1.81 (-2.96, -0.66)** | **0.002** | |
| EQ-VAS | **0.34 (0.18, 0.49)** | **<0.001** | **0.04 (0.02, 0.05)** | **<0.001** | |
| EQ-5D-5L score | **26.98 (14.82, 39.15)** | **<0.001** | **2.23 (0.70, 3.76)** | **0.005** | |
| EQ-5D-dimension mobility (ref. no or slight problems) | -10.54 (-18.43, -2.65) | 0.009 | **-1.06 (-2.03, -0.10)** | **0.031** | |
| EQ-5D dimension Self-care (ref. no or slight problems) | -7.39 (-15.50, 0.71) | 0.073 | -0.68 (-1.66, 0.30) | 0.175 | |
| EQ-5D dimension Daily activities (ref. no or slight problems) | -7.98 (-22.91, 6.95) | 0.292 | -0.79 (-2.59, 1,01) | 0.385 | |
| EQ-5D dimension pain/discomfort (ref. no or slight problems) | **-8.38 (-16.18, -0.58)** | **0.035** | -0.70 (-1.65, 0.25) | 0.150 | |
| EQ-5D dimension Anxiety/depression (ref. no or slightly anxious or depressed) | -7.10 (-14.82, 0.61) | 0.071 | **-1.23 (-2.15, -0.31)** | **0.009** | |
