# Supplementary material for: Care-related quality of life of informal caregivers of stroke survivors: Cross-sectional analysis of a randomized clinical trial
Source: PLoS One. 2024 Oct 4;19(10):e0307930. doi: 10.1371/journal.pone.0307930 (PMC11452055; doi:10.1371/journal.pone.0307930)
Supplement: S2 Table — (DOCX) [file pone.0307930.s003.docx]

**S2 Table.** Internal consistency of the Carer-QoL-7D

|  | **Alpha** | **Item-test correlation** | **Item-rest correlation*** |
| --- | --- | --- | --- |
| Fulfillment | 0.64 | 0.30 | 0.12 |
| Relational problems | 0.59 | 0.53 | 0.34 |
| Mental health problems | 0.51 | 0.72 | 0.55 |
| Financial problems | 0.53 | 0.68 | 0.48 |
| Problems with daily activities | 0.60 | 0.56 | 0.33 |
| Support from others | 0.68 | 0.35 | 0.07 |
| Physical health problems | 0.63 | 0.70 | 0.52 |
| Total scale | 0.63 |  |  |

Alpha: Cronbach’s Alpha values for the scales composed by all items but the one indicated in each row

Item-test correlation: correlation between the item and the total scale

Item-rest correlation: correlation between the item and the scale composed by the rest of items
