# Supplementary material for: Care-related quality of life of informal caregivers of stroke survivors: Cross-sectional analysis of a randomized clinical trial
Source: PLoS One. 2024 Oct 4;19(10):e0307930. doi: 10.1371/journal.pone.0307930 (PMC11452055; doi:10.1371/journal.pone.0307930)
Supplement: Attachment — Submitted filename: Response to reviewers2.docx [file pone.0307930.s005.docx]

Dear Editors and Reviewers of the Plos ONE,

Thank you for the opportunity of resubmitting our work

Hereby we include a one by one answer to the editor and the reviewers’ comments.

1. Regarding data sharing, data for the present analysis was obtained through interviews with stroke patients and their families after informed consent. The informed consent as approved by the Hospital Vall d’Hebron Research Ethics Committee did not include a specific consent for data sharing, thus data cannot be publicly available for ethical reasons.
2. Following the suggestion of reviewer #2 we have added the information related to internal consistency of the CarerQoL-7D

In the methods section, page 7, lines 178-179: “Additionally, we evaluated internal consistency of the CarerQoL-7D in our sample by estimating Cronbach’s Alpha”

In the results section, page 11, lines 241-243: “Cronbach’s Alpha value for the CarerQoL-7D was 0.63, indicating low correlation between items in general, but especially for Fulfillment and Support from others, (all values for item-test and item-rest correlations can be found in supplemental Table S2”

In the discussion section, page 14, lines 297-302: “The fact that internal consistency of the CarerQoL-7D is low indicates that each item is measuring different aspects of care, and that they are not necessarily correlated with each other. This is especially relevant when we intend to measure the positive aspects of care, such as satisfaction or fulfillment, which might be independent from financial or other practical problems. However, it also points out that the overall score of the CarerQoL-7D should be interpreted with caution and taking into account the values for the individual dimensions. “

We have added Table S2 in the Supplemental material:

Table S2. Internal consistency of the Carer-QoL-7D

|  | **Alpha** | **Item-test correlation** | **Item-rest correlation*** |
| --- | --- | --- | --- |
| Fulfillment | 0.64 | 0.30 | 0.12 |
| Relational problems | 0.59 | 0.53 | 0.34 |

| Mental health problems | 0.51 | 0.72 | 0.55 |
| --- | --- | --- | --- |
| Financial problems | 0.53 | 0.68 | 0.48 |
| Problems with daily activities | 0.60 | 0.56 | 0.33 |
| Support from others | 0.68 | 0.35 | 0.07 |
| Physical health problems | 0.63 | 0.70 | 0.52 |
| Total scale | 0.63 |  |  |

Alpha: Cronbach’s Alpha values for the scales composed by all items but the one indicated in each row

Item-test correlation: correlation between the item and the total scale

Item-rest correlation: correlation between the item and the scale composed by the rest of items
